# Recent Advances in Understanding the Inflammatory Response in Malaria: A Review of the Dual Role of Cytokines

**DOI:** 10.1155/2021/7785180

**Published:** 2021-11-08

**Authors:** Gabriela Loredana Popa, Mircea Ioan Popa

**Affiliations:** ^1^Department of Microbiology, “Carol Davila” University of Medicine and Pharmacy, 020021 Bucharest, Romania; ^2^“Cantacuzino” National Military Medical Institute for Research and Development, 011233 Bucharest, Romania

## Abstract

Malaria is a serious and, in some unfortunate cases, fatal disease caused by a parasite of the *Plasmodium* genus. It predominantly occurs in tropical areas where it is transmitted through the bite of an infected Anopheles mosquito. The pathogenesis of malaria is complex and incompletely elucidated. During blood-stage infection, in response to the presence of the parasite, the host's immune system produces proinflammatory cytokines including IL-6, IL-8, IFN-*γ*, and TNF, cytokines which play a pivotal role in controlling the growth of the parasite and its elimination. Regulatory cytokines such as transforming growth factor- (TGF-) *β* and IL-10 maintain the balance between the proinflammatory and anti-inflammatory responses. However, in many cases, cytokines have a double role. On the one hand, they contribute to parasitic clearance, and on the other, they are responsible for pathological changes encountered in malaria. Cytokine-modulating strategies may represent a promising modern approach in disease management. In this review, we discuss the host immune response in malaria, analyzing the latest studies on the roles of pro- and anti-inflammatory cytokines.

## 1. Introduction

Malaria remains a major public health concern and one of the leading causes of death from an infectious disease [1, 2]. In 2019 alone, 229 million cases of malaria were reported worldwide with 409,000 people dying, the majority of whom were children in the African Region [3, 4] Malaria is the most common parasitic disease in the world. It is caused by organisms that belong to the *Plasmodium* genus. The most important transmission route is through the bite of an infected female Anopheles mosquito; the infection can also be acquired through transfusion of infected blood or even transplacentally [5–7]. To date, more than 120 *Plasmodium* species infecting mammals, birds, and reptiles have been identified. Of these, six infect humans (*Plasmodium falciparum*, *Plasmodium vivax*, *Plasmodium ovale wallickeri*, *Plasmodium ovale curtisi*, *Plasmodium malariae*, and *Plasmodium knowlesi*). *Plasmodium falciparum* and *Plasmodium vivax* are the main species responsible for human malaria, causing over 90% of infections [8, 9].

**Figure 1:**
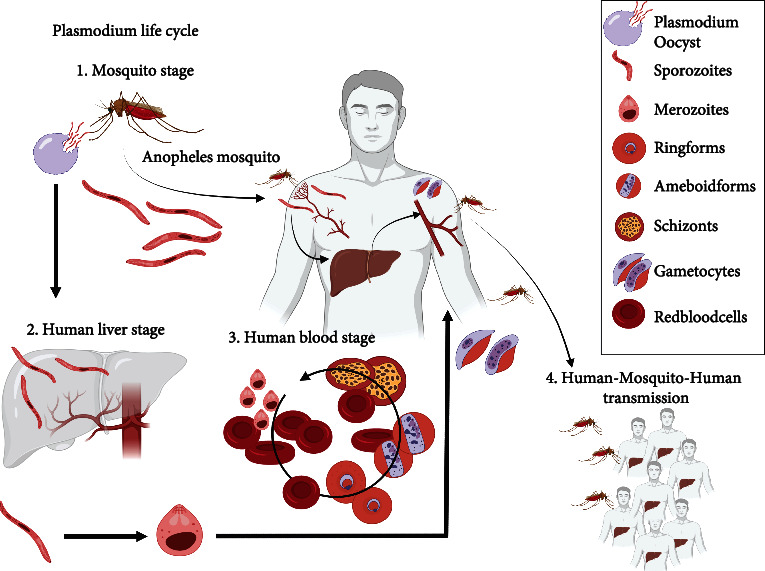
The host-pathogen-environment interactions.

The *Plasmodium* life cycle starts when sporozoites are inoculated in the host subcutaneous capillaries through the bite of an infected female Anopheles mosquito and in about 45 minutes, they reach the hepatocytes [10]. Thus, the first stage of the disease is the preerythrocytic liver stage which lasts 1-2 weeks. In the hepatocytes, sporozoites replicate, resulting in schizonts containing a high number of merozoites. Following the rupture of mature schizonts, the merozoites are released into the bloodstream and diffuse into the body parasitizing the red blood cells. Then, the blood stage is initiated and is characterized by serial cycles of asexual replication: merozoites mature to trophozoites and schizonts, which in turn release new merozoites that will infect other erythrocytes, perpetuating the erythrocyte cycle (Figure 1) [8, 10–12].

Incubation period in malaria is variable, between 7 and 30 days. The infections caused by *Plasmodium* spp. may manifest as asymptomatic parasitemia, uncomplicated malaria, severe malaria, and death [13]. The classical malaria attack is characterized by three stages, the cold, hot, and sweating stages. The clinical picture of malaria includes a large spectrum of unspecific signs and symptoms such as fever, chills, headache, nausea, vomiting, myalgia, arthralgia, and jaundice [11]. In malaria, parasitic antigens along with various host cellular factors induce the release of cytokines from inflammatory cells (macrophages, neutrophils, etc.) and endothelial cells. Elevated levels of cytokines are tied to anemia, liver dysfunction, and fever on the one hand and to parasite control, on the other. Thus, cytokines represent key mediators in the pathogenesis of malaria [14, 15]. In this review, we discuss the host immune response in malaria, analyzing the latest studies on the roles of pro- and anti-inflammatory cytokines.

## 2. Method

We have performed a nonsystematic review using PubMed and Google Scholar databases. We have selected articles written in English published in the last decade, based on relevance. We did not use conventional inclusion and exclusion criteria.

## 3. Effector Cells in Malaria

Innate immunity is the first line of defense against malaria. The inflammatory cells recognize *Plasmodium* PAMPs (pathogen-associated molecular patterns), such as glycosylphosphatidylinositol (GPI), hemozoin, and DNA, via pattern recognition receptors (PRRs) including Toll-like receptors (TLRs), Nod-like receptors (NLRs), RIG-I-like receptors (RLRs), and scavenger receptors (e.g., CD36 and CD204). As a result, a plethora of cytokines and chemokines are synthesized, which contribute to parasite clearance and promote the adaptive immune response [16, 17]. TLRs represent the main receptors involved in the pathogenesis of malaria [18]. MyD88 is an adaptor protein associated with TLR signaling. The activation of MyD88 leads to the recruitment of other signaling molecules and results in the activation of MAPK and NF-*κ*B signaling pathways, which further promote the synthesis of cytokines [19].

GPI is the first molecular compound identified as a PAMP for the malaria parasite. GPI stimulates the release of tumor necrosis factor- (TNF-) alpha and interleukin- (IL-) 1, increases the expression of nitric oxide synthetase, and modulates various signaling pathways resulting in increased expression of ICAM-1, VCAM-1, and E selectin in both inflammatory and endothelial cells. Host immune cells also recognize various endogenous compounds released during the infectious process, known as danger-associated molecular patterns (DAMPs), including high mobility box 1 (HMGB1), HSP70, and SP100 family of proteins [20].

After *Plasmodium* sporozoites enter the human body through mosquito bites, they interact with three main cell populations, specifically CD11c+ antigen-presenting cells, hepatocytes, and Kupffer cells. In hepatocytes, the parasite undergoes changes in its antigenic structure, but it remains unknown which antigens are involved in the activation of CD8+ T cells during this infection stage. Protective CD8+ T cells appear to be activated not just against *Plasmodium* antigens but also against those that are normally expressed only after hepatocyte infection [21]. CD8+ T cells stimulate the production of proinflammatory cytokines (IFN-*γ*, TNF-*α*), which are involved in the generation of nitric oxide that will kill the parasite in the hepatocyte. CD8+ T cells play a central role in the immune response against the preerythrocytic stage of infection [22].

### 3.1. Antigen-Presenting Cells

During the blood infection stage, macrophages and dendritic cells are the first cells to react. After the phagocytosis of infected erythrocytes or merozoites, macrophages become dysfunctional [23]. Recently, it has been shown that the altered macrophage activity is due to phagosomal acidification [24]. However, certain subsets of macrophages such as CD11c+ splenic red pulp macrophages and CD169+ inflammatory macrophages release cytokines [25]. The activated dendritic cells release an abundance of cytokines, especially IL-2, IL-6, and TNF-*α*, that regulate both innate and adaptive immunity [24, 26]. A recent study on mouse models has revealed that *Plasmodium* infection cure cycles could promote a potent recall response against blood-stage parasites, and this finding may be useful in developing new strategies to achieve antimalaria immunity. Dendritic cells of infection-cured mice exhibit fewer MHC II molecules and produce a lower quantity of cytokines following TLR stimulation when compared to uninfected mice [26]. Loughland et al. have shown that CD16+ dendritic cells stimulated by *P. falciparum* might be the only dendritic cell subset activated during primary blood-stage malaria. In addition, these cells seem to contribute to the generation of TNF and IL-10, being involved in both the proinflammatory response and the modulation of immune response [27]. Götz et al. suggest that, in malaria, oxidative stress plays a critical role in increasing the activity of dendritic cells and CD4+ T cells. However, they have shown that activation of T cells by parasite-infected dendritic cells incubated in the presence of xanthine oxidase does not induce altered CD4+ T cell polarization which means that under conditions of oxidative stress, the profile of cytokines released by dendritic cells does not change [28].

### 3.2. Neutrophils

Neutrophils also participate in the protective response against *Plasmodium* spp. in the blood infection stage. They can act through several mechanisms, such as the phagocytosis of blood-stage parasites after antibody opsonization, the generation of oxidative stress, and the formation of reactive oxygen species (ROS), which will hinder intraerythrocytic multiplication [29]. Neutrophil extracellular trap (NET) formation is yet another important mechanism. Crystal uric acid and cytokines, such as TNF-*α* and IL-8, induce NETosis [24, 29]. NETosis is a mechanism by which extracellular parasites are destroyed. Neutrophils release antimicrobial factors. In addition to the beneficial role that neutrophils have in eliminating the parasite, several mechanisms that have been described suggest their involvement in the pathogenesis of severe malaria cases. Products released by neutrophils, such as elastase, exert negative effects on endothelial cells, whilst both TNF-*α* and ROS increase ICAM-1 in which further promotes parasite adhesion [29].

### 3.3. T Cells

In malaria, CD4+ T cells have an immunosuppressive function, inducing a Th1-type immune response that consequently inhibits B cell activity. In addition, dendritic cells modulate and maintain Th1 polarization. Moreover, dendritic cells also promote the activation of natural killer (NK) cells that produce significant amounts of IFN-*γ*, a cytokine strongly associated with a Th1 response [30]. It has recently been shown that adaptive NK cells correlate with malaria characterized by mild symptoms and low parasitemia. Conversely, reduced counts of NK cells correlate with increased mortality and decreased IFN-*γ* production [31].

Costa et al. have evaluated the induction of programmed death-1 (PD-1) and cytotoxic T-lymphocyte antigen (CTLA-4) on regulatory T cells (Treg) in *P. vivax*-infected patients. The authors have observed a high number of circulating Tregs and overexpression of both CTLA-4 and PD-1. They concluded that the regulatory function of Tregs is altered in malaria, and the expression of inhibitory molecules, PD-1 and CTLA-4, is associated with changes in the Treg phenotype, which now display an increased capacity to produce IFN-*γ* compared to other cell subsets [32]. Therefore, *P. vivax*-infected patients have an increased count of activated but exhausted T cells. Blocking CTLA-4 and PD-1 signaling pathways leads to restoring the balance of cytokine production. These results suggest that *P. vivax* modulates the host immune response by inducing the expression of T cell function inhibitory molecules [33].

Recently, the role of *γδ* T cells, a subset of T cells displaying *γδ* cell receptors, has been discussed and it appears that these cells are involved in the production of cytokines and cytotoxicity. *γδ* T cells induce the release of IFN-*γ*, which mediates the elimination of the parasite, but can also directly kill it through their cytotoxic action [34]. In subjects with chronic malaria exposure, it has been shown that the V*δ*2+ subset of *γδ* T cells decreases, which implies the development of an anti-inflammatory response and consequently a clinical tolerance during malaria [35].

### 3.4. B Cells

*P. falciparum* infection elicits a strong inflammatory response and induces the polyclonal activation of B cells along with the production of immunoglobulins. B cell-mediated immune response can be dysfunctional, and they do not acquire long-term immune memory, especially among children in whom the level of antibodies is usually low [36]. Among patients infected with *Plasmodium* spp., a subset of B cells has been identified displaying the transcription factor T bet, known as atypical memory B cells. Their role is unclear, and further studies are needed to establish their contribution to the immune response [36]. These cells may be involved in the delayed and short-lived nature of the humoral immune response against malaria, and their number appears to increase after each acute malaria episode [30]. In contrast, T follicular helper (Tfh) cells play a pivotal role in the formation of long-lived antibodies. A recent study concluded that Th1 cells in malaria can prevent the proliferation of Tfh cells. Thus, in acute malaria, the less-functional Th1-polarized CXCR3+ Tfh subset is activated, whereas the highly functional CXCR3- Tfh subset is not. PD-1+ CXCR5+ CXCR3- Tfh cells have a greater ability to collaborate with B cells than Th1-polarized PD-1+ CXCR5+ CXCR3+ Tfh cells [37].

## 4. Cytokine Network in Malaria

**Figure 2:**
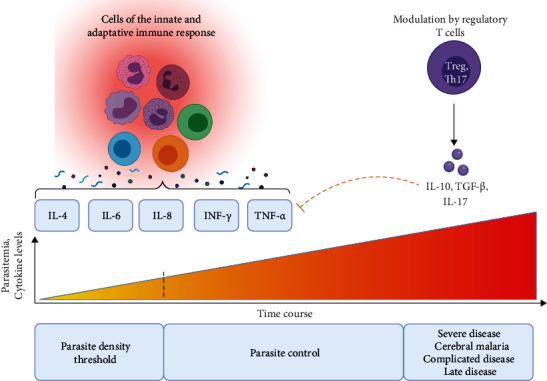
The role of different involved effector cells and cytokine mediators in the pathogenesis of malaria.

During the blood infection stage, in response to the presence of the parasite, the host's immune system releases a number of proinflammatory molecules including IL-1*β*, IL-6, IL-8, IL-12 (p70), IFN-*γ*, and TNF, all cytokines which play a defining role in controlling the parasite's growth and elimination (Figure 2). Regulatory cytokines such as transforming growth factor- (TGF-) *β* and IL-10 maintain the balance between the proinflammatory and anti-inflammatory responses. When this balance is disrupted, the exaggerated proinflammatory response leads to significant adverse effects associated with severe forms of malaria and a high mortality rate [14].

Cytokine production can be influenced by several factors. A recent study has shown that the polymorphism of TLRs affects the synthesis of cytokines in *Plasmodium vivax* malaria. For instance, subjects with the minor alleles of TLR4 (A299G), TLR6 (S249P), and TLR9 (-1486C/T) were detected to have lower amounts of IL-6 and IFN-*γ*. Although these cytokines play a defining role in eliminating the parasite, elevated levels are correlated with disease complications. In addition, the study showed that polymorphisms in the TLR4 (A299G), TLR6 (S249P), and TLR9 (-1486C/T) genes are associated with a lower synthesis of IL-10, an important modulator of the immune response. T/T genotype of the TLR9 polymorphism (-1486C/T) is correlated with elevated levels of IL-2 [18].

Hemmer et al. have shown a more potent host response per parasitized erythrocyte in infections caused by *Plasmodium vivax* or *ovale* than in those attributable by *Plasmodium falciparum* [38]. However, a recent study on vivax malaria has revealed higher IL-10/TNF-*α*, IL-10/IFN- *γ*, and IL-10/IL-6 ratios, but similar inflammatory cytokine responses per parasitized erythrocyte, when compared to falciparum malaria. Additionally, carriers of very low *P. vivax* parasitemia had considerably reduced levels of proinflammatory and regulatory cytokines when compared to patients with clinical manifestations of *P. vivax* malaria [39]. These data draw attention to the fact that reaching the parasite density threshold is probably necessary to activate a host immune response [39].

Scherer et al. suggest that blood viscosity is higher in patients with malaria and ultimately influences cytokine levels. In the serum of patients with *P. vivax* infection, higher levels of IFN-*γ* and IL-17 and lower levels of TGF-*β* were determined. In addition, the study showed that incubation of blood collected from infected patients in the presence of IL-17 or IL-17 and IFN-*γ* led to the normalization of blood viscosity, being similar to that of uninfected individuals [40]. The authors suggest that increasing serum IL-17 levels in malaria patients could be considered a host adaptation mechanism to control changes in blood viscosity, and IL-17 could thus be used as an immunomodulatory agent. IL-17 appears to act on erythrocytes by remodeling their cell membrane; it is well-known that erythrocytes in malaria are very sensitive to osmotic shock [40].

In the following subsections, we present the latest advances made in understanding the role of cytokines in malaria pathogenesis.

### 4.1. TNF-*α*

TNF-*α* plays a pivotal role in increasing phagocytic uptake of parasites, being involved in parasite control [41]. In endemic areas, *P. falciparum*-specific CD4+ T cells coproducing IFN-*γ* and TNF-*α* were detected in patients who underwent *P. falciparum* infection [42]. Consecutively, elevated serum TNF-*α* levels have been reported to directly correlate with the severity of *P. falciparum* malaria. Moreover, recent research states the role of TNF in lethal malaria forms [41]. A systematic review that included 34 studies showed that elevated levels of TNF-*α* could be associated with cerebral malaria caused by *P. falciparum*, but the results are inconsistent. Most studies included a relatively small number of patients; further research is required [43]. In addition, there is a link between polymorphism within the TNF promoter region, parasitemia, and malaria severity. Nguyen et al. have shown that there is a correlation between symptomatic maximum parasitemia and TNF-308, TNF-238, and TNF-244. Furthermore, there is an association between the number of mild malaria episodes and TNF-244 [44]. A recent study presents an interesting finding. The study shows that TNF leads to increased intracellular calcium levels and decreases the count of intracellular parasites, using calcium as the second messenger of the pathway. The same research shows decreased expression of PfPCNA1, which encodes the *Plasmodium falciparum* proliferating-cell nuclear antigen 1, following treatment with TNF [45].

### 4.2. IFN-*γ*

IFN-*γ* plays a dual role in the pathogenesis of malaria. A recent review of the role of IFN-*γ* in malaria suggests that type I IFN signaling limits CD4+ T helper cell activity during the blood infection stage. On the contrary, it is known that type I IFNs stimulate the release of proinflammatory cytokines, participating in the control of infection. The effect of type I IFNs depends on parasite factors as well as host factors [46]. The protective effect occurs especially when the level increases early in the evolution of the infection. However, chronic high levels will often lead to immune suppression [47]. Levels of type I IFNs are influenced by processes such as ubiquitination, phosphorylation, and ADP-ribosylation of the molecules involved in type I IFN pathways. IL-6 has a stimulating effect by increasing STAT1 expression, but there are also several other compounds with immunosuppressive effects, including suppressor of cytokine signaling 1 and 3 and ubiquitin carboxy-terminal hydrolase 18. In addition, molecules in the parasite structure may exert a regulatory effect on host type I IFN response [47]. Lourembam et al. have analyzed 58 patients infected with *P. falciparum* who had been divided according to WHO criteria into two groups with complicated and uncomplicated malaria. In the complicated malaria lot, higher levels of IFN-*γ* and TGF-*β* and lower levels of IL-2 and IL-12a were reported when compared to the uncomplicated malaria cases. Moreover, the authors suggest that T cells are not a source for elevated levels of IFN-*γ* and draw attention to the need for further investigation of cells responsible for the exaggerated production of IFN-*γ* in cerebral malaria [48].

### 4.3. IL-6 and IL-8

IL-6 and IL-8 are proinflammatory cytokines that may be involved in the pathogenesis of malaria. Sebina et al. have shown that IL-6 has several roles in malaria; IL-6 participates in immunoglobulin synthesis, promotes the expression of inducible T cell costimulator (ICOS) in the Tfh cells, and activates the differentiation of B cells. Although IL-6 is an important factor of humoral immunity, the authors suggest that IL-6 appears to be nonessential in the control of *Plasmodium* infection and is only involved in the early stages of infection [49]. Mbengue et al. consider that elevated levels of TNF-*α* and IL-6 could be regarded as markers for severe malaria [50]. Moreover, elevated levels of IL-8 have been identified in malaria patients, and a correlation between IL-8 and disease severity has also been noted. *P. falciparum* has the ability to produce a functional histamine-releasing homologous factor that promotes IL-8 release from neutrophils [50]. It has been reported that IL-8-251T/A promoter polymorphism is correlated with an increased risk of developing complicated malaria [51]. Rodrigues-da-Silva et al. have revealed elevated levels of TNF-*α*, IFN-*γ*, IL-6, IL-8, IL-10, and IL-17 during the acute phase of malaria, and the high levels persisted through convalescence with the exception of IL-10 [52]. According to a study performed by Otterdal et al., IL-27 levels are higher in patients with malaria than in healthy subjects. A positive correlation was also observed between IL-27, *P. falciparum* parasitemia, and von Willebrand factor, but no impact on disease outcome was observed. IL-27 could be seen as an immunoregulatory cytokine in malaria, having a proinflammatory (induces IL-6 release) as well as an anti-inflammatory (inhibits IL-8 release) role [53].

### 4.4. IL-10

IL-10 can be both a friend and an enemy, depending on the immune response and the type of parasite [54]. Nakamae et al. have revealed that IL-10 inhibits protective immune responses against secondary infection with heterologous *Plasmodium* parasites. They have used infection models of *P. chabaudi chabaudi* (Pcc) and *P. berghei* ANKA (PbA) to observe the inhibition of IL-10 in association with increased CD4+ T cell activity, the release of IFN-*γ*, and decreased parasitemia [54]. A recent study found a correlation between *P. falciparum*-specific IL-10-positive T cells (IFN-*γ*- TNF-) and the risk of clinical malaria once infected [55]. Requena et al. have compared the frequencies of T cell subsets among pregnant and nonpregnant women in a malaria-endemic area versus in a malaria-free zone. They have demonstrated that nonspecific proinflammatory responses at the first antenatal visit were associated with protection against *P. falciparum* malaria at delivery. Elevated intracellular levels of IL-10 in CD4+ T cells had a protective effect against *P. falciparum* infection and on hemoglobin levels at delivery [56]. The plasma levels of IL-10/TNF-*α* and IL-10/IFN-*γ* ratios correlate with *P. vivax* concentrations in the blood [57]. Sukhbaatar et al. have revealed that IL-10 production in CD4+ T cells is modulated by IL-27 in chronic *Plasmodium chabaudi* infection and have focused on the pivotal role of the two cytokines in modulating pro- and anti-inflammatory responses in *Plasmodium* infections [58]. *Plasmodium* may promote decreased IL-10 expression by downregulating GATA3 expression, resulting in poor control of the inflammatory process [59].

### 4.5. IL-4

The role of IL-4, an important regulator of Th2 responses, is incompletely elucidated in malaria. IL-4 induces Th2 responses and limits both the inflammatory process and Th1 responses, being associated with a protective role in severe forms of malaria [60]. Wu et al. have evaluated the therapeutic potential of IL-4 in severe malaria in *Plasmodium berghei* ANKA-infected mice. IL-4 treatment has led to reduced parasitemia and decreased mortality. Several mechanisms could be involved, including increased receptor expression for phagocytosis, high IgM synthesis, and decreased inflammatory processes in the brain [61]. However, it should be taken into consideration that, as in the case of the cytokines discussed above, the role of IL-4 depends on certain factors. It has been highlighted that dendritic cells release IL-4 early during malaria and in this case, IL-4 may contribute to the development of a severe form of malaria [60]. Elhussein et al. emphasized that IL-4 could be considered a risk factor for severe forms of malaria and that there is a positive correlation between IL-4 levels and parasitemia [62].

### 4.6. TGF-*β*

TGF-*β* has an anti-inflammatory effect by inhibiting Th1 cell differentiation and thus blocking the production of Th1-derived IFN-*γ*. TGF-*β* also inhibits Th2 cell differentiation, but this subset of cells does not appear to be involved in malaria [63]. Several studies have shown a negative correlation between TGF-*β* levels and the severity of *P. falciparum* infection [64, 65]. de Jong et al. have analyzed the levels of several cytokines in a group of 15 volunteers before and during (at the day of treatment) a controlled malaria infection. They have reported that 9 subjects had low levels of TGF-*β* and high levels of IFN *γ*, IL-6, D dimer, and von Willebrand factor when compared to their respective baseline status. In contrast, in 6 subjects, the authors recorded elevated levels of TGF-*β*, with no changes in the other evaluated parameters, and among these patients, the symptoms were less severe in comparison to the other group [66]. Keswani and Bhattacharyya have shown that the interplay between TGF-*β* and TNF-*α* is involved in splenocyte apoptosis in experimental brain malaria. Furthermore, they have observed that inhibition of TNF-*α* leads to a delay in splenocyte apoptosis, suggesting there probably is a competition between the two cytokines during infection [67]. TGF-*β* and IL-6 modulate the function of several immune cells after a malaria infection, including dendritic cells, regulatory T cells, and T-helper cells (Th17) [68]. According to a recent review, TGF-*β* is involved in the expansion of FoxP3 Tregs, an important mechanism to limit the inflammatory process in malaria [63].

### 4.7. Other Cytokines

de Menezes et al. have shown that neutrophils are an important source of IL-1*α* in the liver during malaria infection. IL-1*α* deficiency has been associated with lower levels of TNF-*α*, weight loss, and hypothermia but bears no significant effects on parasitemia. The authors emphasized the potential role of IL-1*α* in the hepatic inflammatory process [69]. Kisia et al. were the first to demonstrate the correlation between genotypes and haplotypes of IL-7 (72194 T/C and -2440A/G) and inefficient erythropoiesis [70]. A recent study showed that IL-12 and IL-18 play a central role in modulating the innate immune response in malaria. The synergistic effect of IL-12 and IL-18 seems to be involved in *γδ* T cell immunoregulation. The two cytokines are required for the upregulation of the inhibitory receptor TIM3, expressed by *γδ* T cells. The overexpression of TIM3 was associated with a decreased risk of clinical malaria [71]. In a group of patients infected with *P. vivax*, Costa et al. have found thrombocythemia and low levels of IL-2 and IL-12 that correlated with platelet distribution width (PDW) values and concluded that these changes may represent an immune response to thrombocytopenia [72].

Aljarba et al. have reported that IL-22 polymorphisms in rs2227481 and rs2227483 are involved in the mounting of a protective immune response against *Plasmodium falciparum* infection. The authors also point out that the G allele of rs2227513 plays an important role in the increased production of IL-22 [73]. A recent study highlighted the role of IL-35 in malaria. Bello et al. have identified that IL-35 is overexpressed in the serum and tissues of *P. berghei*-infected mice. In addition, there was a positive correlation between the levels of IL-35 and parasitemia. IL-35 neutralization has been associated with beneficial effects on parasitemia, tissue histological changes, and even with a higher survival rate in *P. berghe*i-infected mice (Table 1) [74].

## 5. Conclusions

Based on the conclusions reached in the aforementioned studies, we gather that cytokines are important modulators of the immune response in malaria. Dysregulation of the cytokine network in severe malaria is linked to parasite and host factor variations. We have found that many of the cytokines involved in malaria (TNF-*α*, IFN-*γ*, IL-4, and IL-10) play a double role, as a friend or as an enemy. Proinflammatory cytokines control parasite multiplication and promote parasite clearance. However, elevated levels of proinflammatory cytokines such as TNF-*α*, IL-6, or IL-8 may be markers of severe malaria. TGF-*β* is probably the most important regulatory cytokine that limits the inflammatory process in malaria. Maintaining a balance between proinflammatory and anti-inflammatory cytokines is essential, and disrupting this molecular harmony can lead to unfavorable disease evolution. A better understanding on the cytokine's involvement in malaria pathogenesis could provide the basis for the discovery of novel diagnostic markers and indicators of disease progression and severity, as well as the foundation for the development of new malaria vaccines.

## Figures and Tables

**Table 1:** The role of cytokines in the disease pathogenesis.

Cytokine	Role in the pathogenesis of malaria
TNF alpha	Increases phagocytic uptake of parasites
	Elevated levels correlate with the severity of malaria
	May be associated with cerebral malaria
	Increases intracellular calcium levels and decreases the count of intracellular parasites

IFN gamma	Type I IFN signaling limits CD4+ T helper cell activity during the blood infection stage
	Type I IFNs stimulate the release of proinflammatory cytokines
	Participates in the control of infection
	Chronic high levels may lead to immune suppression

IL-6	Participates in immunoglobulin synthesis
	Promotes the expression of ICOS in the Tfh cells and activates the differentiation of B cells
	Is only involved in the early stages of infection
	Could be regarded as marker for severe malaria
	Its levels increase during the acute phase of malaria that persist through convalescence

IL-8	Correlates with disease severity
	Its levels increase during the acute phase of malaria that persist through convalescence

IL-10	Inhibits protective immune responses against secondary infection
	Its inhibition is associated with increased CD4+ T cell activity, the release of IFN-*γ*, and decreased parasitemia
	*P. falciparum*-specific IL-10-positive T cells (IFN-*γ*- TNF-) correlate with the risk of clinical malaria
	Elevated intracellular levels in CD4+ T cells have a protective effect against *P. falciparum* infection and on hemoglobin levels at delivery

IL-4	Is an important regulator of Th2 responses
	Limits both the inflammatory process and Th1 responses
	Has a protective role in severe forms of malaria
	Could be considered a risk factor for severe forms of malaria

TGF-beta	Has an anti-inflammatory effect by inhibiting Th1 cell differentiation
	Negatively correlates with the severity of *P. falciparum* infection
	Modulates the function of several immune cells after a malaria infection, including dendritic cells, regulatory T cells, and T-helper cells (Th17)
	Is involved in the expansion of FoxP3 Tregs
